# O9-6 Shedding Light on men's health: Evaluating the impact and scalability of a community-based men's health promotion programme “Sheds for Life” in Irish Men's Sheds

**DOI:** 10.1093/eurpub/ckac094.070

**Published:** 2022-08-29

**Authors:** Aisling McGrath, Niamh Murphy, Noel Richardson, Edel Byrne

**Affiliations:** 1 Department of Sport and Exercise Science, Waterford Institute of Technology, Waterford, Ireland; 2 healthCore, Institute of Technology Carlow, Carlow, Ireland; 3 Health and Wellbeing, Irish Men's Sheds Association, Dublin, Ireland

**Keywords:** men's health, health promotion, implementation science, public health interventions

## Abstract

**Background:**

Sheds for Life (SFL) is a ten-week health and wellbeing programme delivered in the community setting of Men's Sheds in Ireland that uses gender-sensitive approaches to engage typically hard-to-reach men (?Shedders') with health. SFL consists of a health check, core modules of physical activity (consisting of a walking programme or chair-based exercises for older adults), mental wellbeing, healthy eating and other elective wellbeing components. SFL is implemented across two phases with four regions per phase. The purpose of the research is to evaluate SFL using an implementation science approach to assess programme impact and implementation effectiveness with a view to enhancing its sustainability and scalability while also informing gender-sensitive strategies that engage hard-to-reach men with health and wellbeing.

**Methods:**

This study is a hybrid typology ?effectiveness-implementation? design. A community-based participatory research, and mixed methods approach has been adopted to measure the effects of the SFL intervention on Shedders across implementation phases and identify and monitor implementation barriers and facilitators that can inform future sustainability and scale-up of SFL. Central to effective implementation of SFL is a partnership approach between the Irish Men's Sheds Association (IMSA) and other health-related partner organisations (POs). This research engages key stakeholders (at individual (Shedder), provider (POs) and organisational (IMSA) levels) and prioritises implementation outcomes. Purposive sampling is used to recruit a diverse sample of participants (Shedders n = 420 and SFL providers n = 20).

**Results:**

Findings from phase 1 will inform phase 2 implementation. Preliminary physical activity outcome results from phase one across four regions (baseline to ten weeks) suggest days active per week increased from 3.07 to 4.32 days (p =.00) days walking increased from 4.29 to 5.28 days (p=.00) minutes walking per day increased from 33.31 to 38.15 (p=.005)

**Conclusions:**

Preliminary findings highlight the potential of the SFL initiative to address the increasing calls for gender-specific health promotion programmes that target lifestyle and health behaviour change in men. Shed settings are unique and effective in attracting men from more marginalised male subpopulations, reaching men who would typically not engage with health services.

This study is funded by the Irish Research Council (ID: EBPPG/2018/256)

